# A novel antegrade biopsy approach using a 3-o'clock-channel cholangioscope with a metal stent mesh laser ablation access technique

**DOI:** 10.1055/a-2888-0564

**Published:** 2026-06-24

**Authors:** Takeshi Ogura, Kimi Bessho, Junichi Nakamura, Nga Nguyen Trong, Hiroki Nishikawa

**Affiliations:** 1 Pancreatobiliary Advanced Medical Center 38588 Osaka Medical and Pharmaceutical University HospitalTakatsukiJapan; 2 Endoscopy Center 38588 Osaka Medical and Pharmaceutical University HospitalTakatsukiJapan; 3 2nd Department of Internal Medicine 13010 Osaka Medical and Pharmaceutical UniversityTakatsukiOsakaJapan; 4 Department of GastroenterologyTrong Nam Cancer HospitalHà NộiViet Nam

In patients with surgically altered anatomy and duodenal stenosis, histological diagnosis under endoscopic retrograde cholangiopancreatography guidance is often difficult. Although endoscopic ultrasound (EUS)-guided antegrade biliary access is an alternative, antegrade biopsy of distal biliary strictures remains challenging due to acute B2–B3 angulation. 1​ ​2​ Cholangioscopy-guided biopsy may overcome this limitation, but conventional scopes deliver biopsy devices from the 6-o’clock position, hampering precise axis alignment in the antegrade setting. A newly developed cholangiopancreatoscope with a 3-o’clock working channel exit (Briview, SeeGen Co., Ltd, Shanghai, China) may facilitate antegrade cholangioscopic biopsy. Because cholangioscope insertion via an EUS-guided hepaticogastrostomy (HGS) requires a mature fistula, technical tips for a novel antegrade biopsy using a 3-o’clock-channel cholangioscope with a metal stent mesh laser ablation technique are described.

**Fig. 1:**
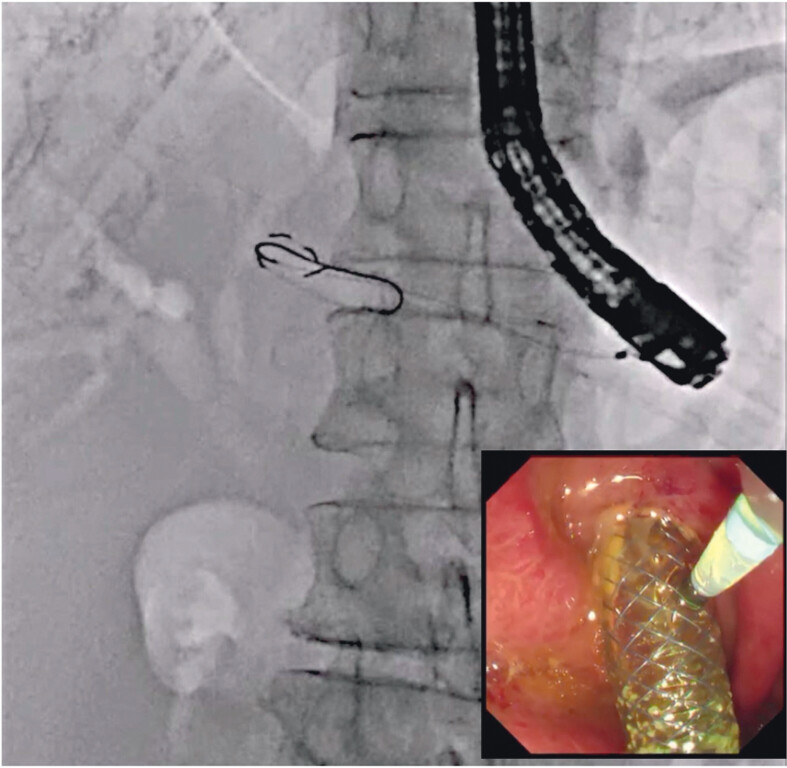
A guidewire is deployed through the mesh of the endoscopic ultrasound-guided hepaticogastrostomy stent.

**Fig. 2:**
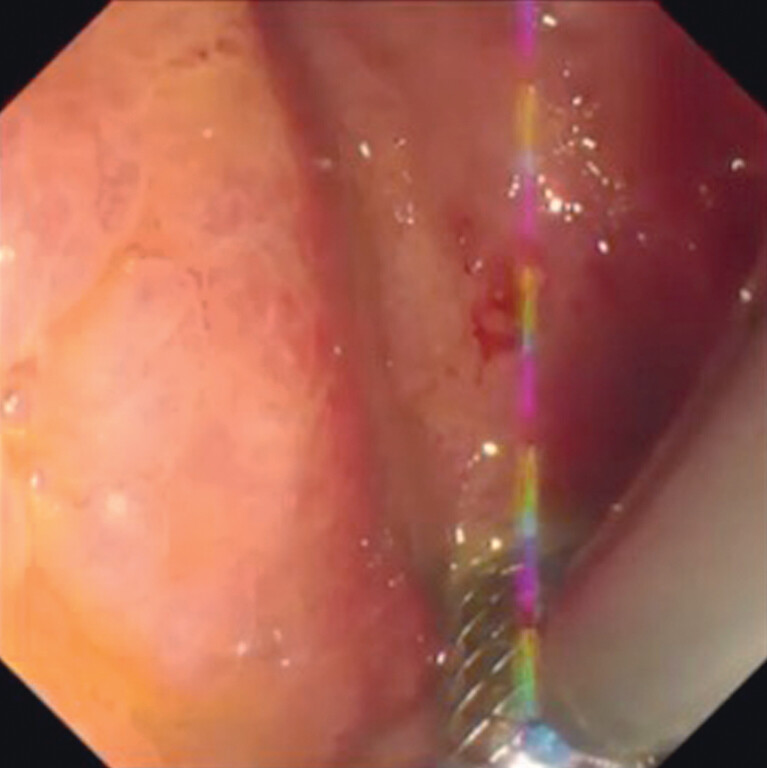
The mesh of the endoscopic ultrasound-guided hepaticogastrostomy stent is broken by laser ablation under cholangioscopic guidance.

**Fig. 3:**
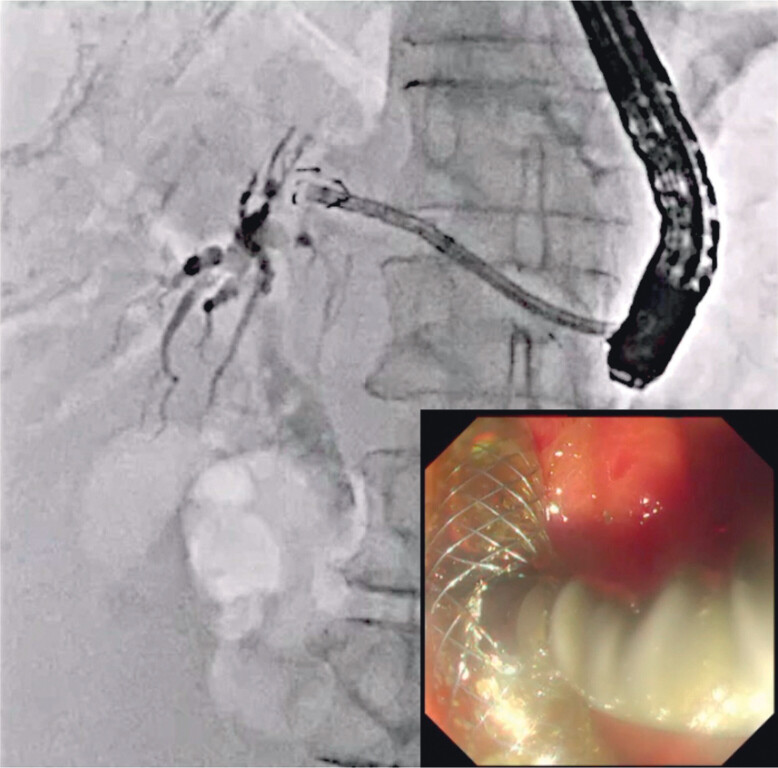
Cholangioscope insertion into the biliary tract through the mesh of the endoscopic ultrasound-guided hepaticogastrostomy stent is performed.

**Fig. 4:**
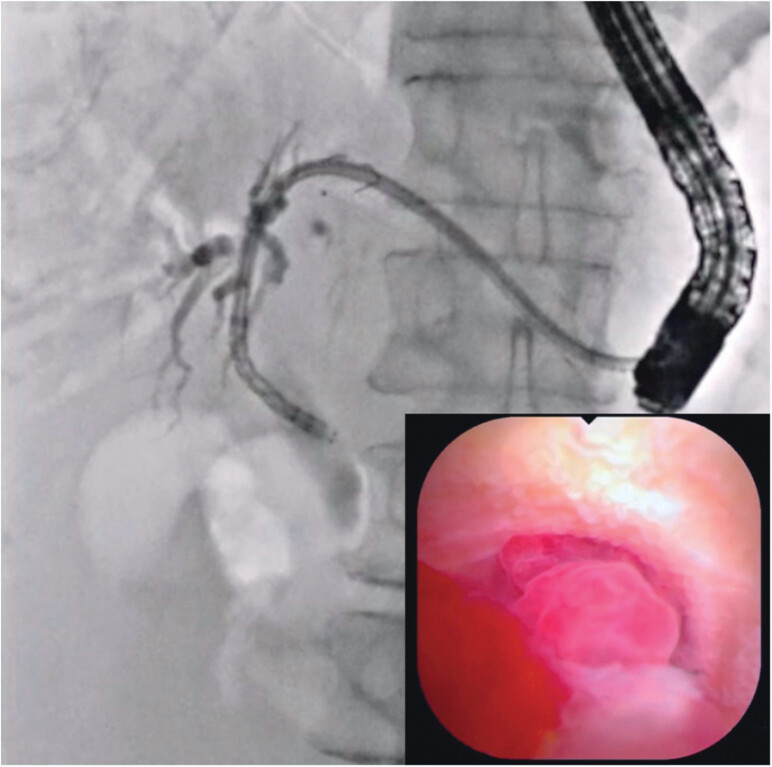
The tumor lesion can be observed.

**Fig. 5:**
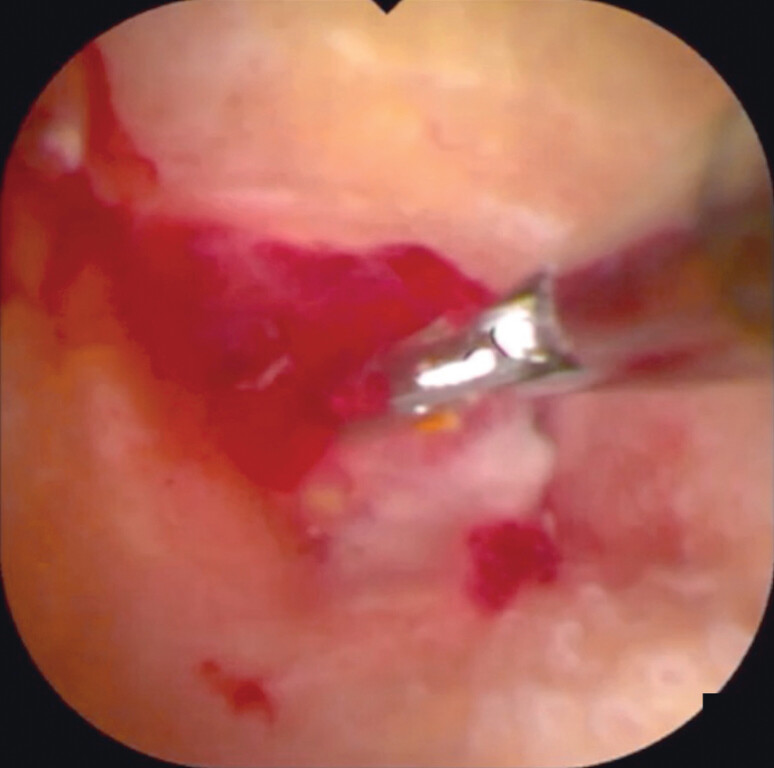
Antegrade biopsy can be easily performed because the biopsy device is extracted from the 3-o’clock position.

An 81-year-old man was admitted with obstructive jaundice. Endoscopic passage through the duodenum was impossible due to tumor-related extrinsic compression, and biopsy from the compressed site showed no malignancy. Therefore, EUS-HGS using a covered self-expandable metal stent was initially performed. Two days later, a duodenoscope was inserted. First, a guidewire was deployed through the mesh of the HGS stent ( Fig. 1 ). Because a mature fistula may not be created, the mesh of the HGS stent was broken by laser ablation under cholangioscopic guidance ( Fig. 2 ). Laser ablation was performed using a holmium laser system (Quanta Litho Evo Laser; Quanta System, Milan, Italy) at a dedicated setting (1J, 10Hz, and 10W). Subsequently, the cholangioscope was successfully inserted into the biliary system through the broken HGS stent ( Fig. 3 ). The cholangioscope was also successfully advanced into the common bile duct, and the tumor was identified ( Fig. 4 ). A target biopsy could be easily performed because the biopsy device was extracted from the 3-o’clock position ( Fig. 5 ) without any adverse events ( Video 1 ). This patient was finally diagnosed as having advanced cholangiocarcinoma.

**Video 1** Antegrade cholangioscopy through the mesh of an endoscopic ultrasound-guided hepaticogastrostomy stent is performed.


In conclusion, antegrade biopsy using a 3-o’clock-channel cholangioscope with a metal stent mesh laser ablation access technique might be helpful for selected patients.

Endoscopy_UCTN_Code_TTT_1AR_2AL
